# Recommendations for conducting the rodent erythrocyte *Pig‐a* assay: A report from the HESI GTTC *Pig‐a* Workgroup

**DOI:** 10.1002/em.22427

**Published:** 2021-03-02

**Authors:** Stephen D. Dertinger, Javed A. Bhalli, Daniel J. Roberts, Leon F. Stankowski, B. Bhaskar Gollapudi, David P. Lovell, Leslie Recio, Takafumi Kimoto, Daishiro Miura, Robert H. Heflich

**Affiliations:** ^1^ Litron Laboratories Rochester New York USA; ^2^ Covance Laboratories Inc. Greenfield Indiana USA; ^3^ Charles River Laboratories Skokie Illinois USA; ^4^ Toxicology Consulting Midland Michigan USA; ^5^ St. George's University of London London UK; ^6^ Integrated Laboratory Systems Research Triangle Park North Carolina USA; ^7^ Teijin Pharma Limited Hino Tokyo Japan; ^8^ U.S. Food and Drug Administration/NCTR Jefferson Arkansas USA

**Keywords:** flow cytometry, genotoxicity, mutagen, mutation assay, *Pig‐a* gene

## Abstract

The rodent *Pig‐a* assay is a flow cytometric, phenotype‐based method used to measure in vivo somatic cell mutation. An Organization for Economic Co‐operation and Development (OECD) test guideline is currently being developed to support routine use of the assay for regulatory purposes (OECD project number 4.93). This article provides advice on best practices for designing and conducting rodent *Pig‐a* studies in support of evaluating test substance safety, with a focus on the rat model. Various aspects of assay conduct, including laboratory proficiency, minimum number of animals per dose group, preferred treatment and blood sampling schedule, and statistical analysis are described.

## 1 INTRODUCTION

The phosphatidylinositol glycan, class A (*Pig‐a*) gene codes for an enzyme that is essential for glycosylphosphatidylinositol (GPI) anchor biosynthesis (Miyata *et al*., 1993). Thus, inactivating *Pig‐a* mutations result in cells that lack cell surface GPI anchors, and as a consequence, GPI‐anchored protein(s); this phenotype represents a reliable reporter of *Pig‐a* mutation in vivo (Kimoto *et al*., 2011b; Revollo *et al*., 2018, 2019, 2020; Dad *et al*., 2020). The analytical approach used to perform these assays utilizes fluorescently conjugated antibodies against GPI‐anchored cell surface epitopes, which makes it possible to measure mutant cell frequencies via flow cytometry (reviewed by Gollapudi *et al*., 2015).

Rodent studies have focused on measuring mutations using erythrocytes, as these cells are easily obtained in sufficient quantity via small volume blood draws. The low blood volume requirement, option for multiple blood draws without euthanizing animals, compatibility with commonly used rodent models, and relatively low cost of these studies in comparison to other in vivo mutation test systems, all make the *Pig‐a* assay attractive for studies of somatic cell mutations (Schuler *et al*., 2011; Gollapudi *et al*., 2015).

The erythrocyte‐based *Pig‐a* assay is considered useful for regulatory safety assessments. For example, as described by the International Council for Harmonization of Technical Requirements for Pharmaceuticals for Human Use (ICH) M7(R1) Guideline on the Assessment and Control of DNA Reactive (Mutagenic) Impurities in Pharmaceuticals (ICH, 2017), the rodent *Pig‐a* assay is one of the recommended follow‐up tests to a positive bacterial mutagenicity finding. This and other use cases have led to efforts to develop an Organization for Economic Cooperation and Development (OECD) test guideline to support regulatory safety assessment studies (OECD project number 4.93).

Several laboratories are establishing proficiency with the assay ahead of test guideline acceptance, and in some cases conduct rodent *Pig‐a* studies in order to generate supplemental information for regulatory approval packages. We have therefore developed these recommendations with the goal of providing stakeholders with current thinking and best‐practices advice regarding laboratory training, study design, and implementation of rodent *Pig‐a* studies. The minimum number of animals per dose group, a preferred treatment and blood harvest schedule, statistical analysis, and other considerations are described. More detailed information on the analytical procedures involved with conducting the assays can be found elsewhere (Kimoto *et al*., 2011a; Dertinger *et al*., 2011b; Bemis *et al*., 2019; Chikura *et al*., 2019; Dobrovolsky *et al*., 2020 ; Chikura *et al*., 2021). The advice provided herein has been designed to serve the needs of the genetic toxicology community as they contemplate establishing laboratory proficiency and/or conducting these studies.

## 2 IN‐LIFE AND ANALYTICAL SITE CONSIDERATIONS

### 2.1 In‐life facility

The in‐life portion of the test should be conducted at a site where work is overseen by an Institutional Animal Care and Use Committee (IACUC), an Animal Welfare and Ethical Review Body (AWERB), or a local equivalent. This oversight ensures that experiments utilizing vertebrate animals have merit, animal welfare standards are met, staff have been trained on all necessary procedures, and all aspects of the work are sufficiently supervised.

Standard housing, bedding, enrichment, feed and water schedules should be employed, and animals should be group housed unless exceptions are scientifically justified (e.g., aggression, or endpoint specific requirements when the *Pig‐a* assay is integrated into a repeat‐dose general toxicology study). As discussed in more detail below, some studies may involve only one sex, while other studies are conducted with both sexes.

One of the major advantages of the *Pig‐a* gene mutation assay is that it can be performed with transgenic animals as well as more widely available, non‐transgenic laboratory rodent models (Shemansky *et al*., 2019). This facilitates the use of the most appropriate species/strain when evaluating mutagenicity in vivo, a decision that may be influenced by pharmacodynamic, pharmacokinetic, tolerability, or bioanalytical data. While this flexibility is clearly beneficial, it is important for the in‐life facility to have prior experience with each specific rodent strain being contemplated for a definitive study. As explained below, this is because each regulatory study should have an accompanying historical negative control database for the animal model used.

The majority of rodent *Pig‐a* experiments conducted for regulatory safety assessment are expected to involve either stand‐alone or integrated study designs that consist of 28‐consecutive days of dosing. Laboratories, therefore, should have adequate staff to ensure the dosing schedule is maintained without interruption, and that the health of animals is monitored regularly. Veterinary staff and the Study Director/Principal Investigator must be present physically or available on‐call throughout the duration of the in‐life phase in case prompt decisions about treatment (e.g., dose suspension, discontinuation, or adjustment) need to be made due to unexpected morbidity or mortality.

In many cases, red blood cell labeling and flow cytometric analyses are conducted at the same facility that conducts the in‐life phase of the study. However, we have separated the in‐life phase from the flow cytometric analysis phase to emphasize that these functions can be performed at different facilities. Briefly, as described in greater detail below, anti‐coagulated blood samples that are kept cold (~ 4°C), not frozen, throughout transportation are compatible with analysis. Furthermore, procedures for freezing blood samples have been described (Avlasevich *et al*., 2019), and these can be used for shipping frozen samples to a separate analytical facility, provided samples remain frozen throughout transportation.

### 2.2 Analytical facility

Each facility analyzing the blood samples should be able to provide assurance that proficiency demonstrations have been successfully completed and that historical negative control databases have been generated for the animal model being considered for a regulatory safety assessment study. It is important for the staff to have demonstrated proficiency with sample processing and analysis. Based on the authors' collective experience with training numerous personnel across different laboratories, we recommend a three‐step process.

After personnel have been introduced to the necessary blood sample processing and flow cytometric analysis procedures, the first key set of recommended proficiency experiments are reconstruction or “spiking” experiments (for details, see Raschke *et al*., 2016). Briefly, a single rodent is exposed to a known, potent mutagenic substance, *for example*, *N*‐ethyl‐*N*‐nitrosourea (ENU). After an appropriate phenotypic expression time that allows elevated mutant reticulocyte (MUT RET) and mutant erythrocyte (MUT RBC) frequencies to appear in the peripheral blood compartment, blood from the exposed rodent and a sex/age‐matched negative control animal (either naïve or vehicle treated) should be collected. (Note: hereafter, “MUT RET/RBC” is used to indicate both MUT RET and MUT RBC.) The two blood samples are combined in a series of serial dilutions to create a range of MUT RET/RBC frequencies (i.e., spiked samples). After determining MUT RET/RBC frequencies separately for the mutagen‐treated and the negative control animal, expected intermediate frequencies can be calculated for the spiked samples based on the proportion of blood from the mutagen‐exposed animal added to the negative control blood. By conducting reconstruction experiments on several separate occasions, and with several replicates per spiked sample, staff can be trained on important elements of the assay by using a minimal number of animals. The proficiency of staff members is established by demonstrating agreement between the observed and expected MUT RET/RBC frequencies (Raschke *et al*., 2016). Successful completion of spiking experiments represents a useful, 3Rs‐friendly gateway to further proficiency investigations as described below.

The second step requires the laboratory to reproduce expected results from high quality, peer‐reviewed data (as collected in the on‐line *Pig‐a* database described in Shemansky *et al*., 2019). This should be demonstrated for MUT RET/RBC frequencies using a minimum of two well‐established mutagenic substances. These experiments should use doses that give reproducible and dose‐related increases in mutant frequencies and demonstrate the sensitivity and dynamic range of the test system. Mutagenic agents that have been studied at multiple laboratories for this purpose include, but are not limited to: ENU, 7,12‐dimethylbez[*a*]anthracene (DMBA), 4‐nitroquinoline 1‐oxide (4‐NQO), melphalan, thiotepa, 1,3‐propane sultone, procarbazine, and chlorambucil.

As with the previous two steps, the third step should be accomplished before the first definitive study occurs—that is, a historical negative control database should be established for each species/strain that will be used. One tip for efficiently developing historical negative control databases is to collect and analyze pre‐dosing (i.e., “baseline”) blood samples, for example from rodents used in the step 2 proficiency experiments, provided the methodology was consistent with what will be used for future studies and sample processing was technically proficient. Note that the ability to construct historical negative control databases with naïve animals in combination with those treated with common vehicles (e.g., sesame oil, olive oil, water, 0.9% saline, phosphate buffered saline, and methylcellulose/aqueous solutions) stems from the equivalence of their MUT RET/RBC frequencies (OECD, 2020a).

The development of historical negative control databases also benefits from the fact that while rat reticulocyte frequencies (i.e., %RET) tend to be influenced by sex and age, no significant differences in negative control MUT RET/RBC frequencies have been detected between rodents that differ in age by several months (OECD, 2020a). Thus, it is possible to use a range of ages for building historical negative control MUT RET/RBC databases provided this variable is tracked and periodically reconsidered for its influence on MUT RET/RBC frequencies.

Based on the literature, it should be acceptable to initially consider sex as having no effect on negative control MUT RET/RBC frequencies from young, healthy rodents (Labash *et al*., 2015). Thus, historical negative control distributions can initially be constructed using animals of either sex, or both sexes combined. However, similar to the age variable, laboratories should periodically reconsider the assumption that sex has no influence on MUT RET/RBC frequencies by testing for an effect using the data for males and females in the database. As long as sex is not found to be a significant factor, the MUT RET/RBC distributions can be assembled by combining data from both male and female animals. If sex differences are observed, this would indicate that sex‐specific historical negative control databases are appropriate for this rodent model. Likewise, data collected from different rodent strains should be tracked in a similar fashion and pooled only if there are no statistical differences between the MUT RET/RBC data distributions.

When first acquiring data for inclusion in the historical negative control database, they should be consistent with published data (Shemansky *et al*., 2019). As more experimental data are added to the historical control database, MUT RET/RBC frequencies from individual naïve and/or vehicle control animals should be free from known technical error and *ideally* fall below the upper bounds of the existing historical negative control distribution (see below for exceptions). Various distribution models are acceptable and should be internally justified prior to use. Generally speaking, “observed range” (i.e., lowest to highest observed frequencies) is not useful for describing the historical negative control distribution except when the number of individual animals studied is very low (e.g., *n* < 30). Once sufficient numbers of animals are included in the database, other approaches for characterizing the historical negative control distribution are preferred, for example, 95% control limits, 99% control limits, prediction intervals, and tolerance intervals (Vardeman, 1992). Prior to implementing a model for ascertaining distribution limits, one should ensure *a priori* requisites such as normality are satisfied. Data transformation(s) can be valuable for this purpose.

Note also that by definition, a small proportion of MUT RBC/RET frequencies are expected to fall outside of an existing historical negative control distribution, and that all technically valid data should be included to accurately represent negative control MUT RET/RBC frequencies. That being said, given the clonal nature of mutation, an extreme high outlier can be expected on rare occasions. For instance, the authors have observed rare naïve mice and rats to have hundreds or even thousands of mutant cells per million. In these cases, when an individual single animal's mutant frequency markedly distorts historical negative control distribution metrics, it will often be appropriate to omit the individual from the database.

The laboratory's historical negative control database should be adequate for assessing the acceptability of negative control data in a definitive study. Therefore, as a starting point, each laboratory should acquire MUT RET/RBC frequency measurements from at least 30 naïve and/or vehicle‐treated animals from each rodent strain used for testing. Each MUT RET/RBC frequency value should be acquired from an individual animal. Thus, multiple serial blood samples from the same animal should not be added to the historical negative control database. Finally, the data should be acquired from at least three independent experiments that each use progeny from different breeding cycles.

**FIGURE 1:**
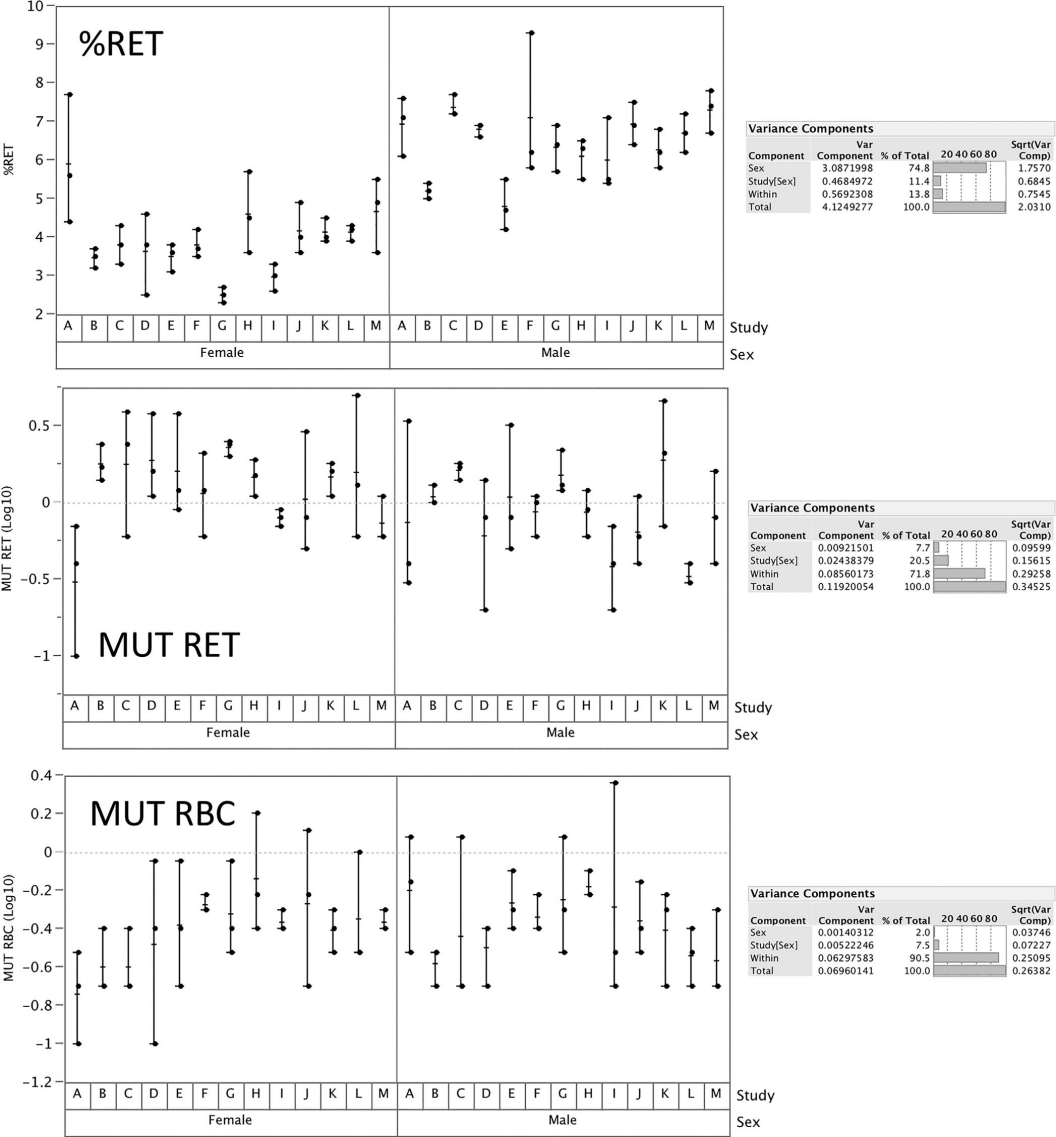
Reticulocyte (RET), and mutant reticulocyte (MUT RET) and mutant erythrocyte (MUT RBC) frequencies are graphed for male and female rats (39 each) that had been exposed to one of several common vehicles. Blood samples were collected when the rats were 7 weeks old. Whereas each circle represents an individual animal, the ranges are denoted by the length of horizonal lines, and group means are indicated by a vertical tick mark. These data were evaluated by Restricted Maximum Likelihood analysis and demonstrate that variation in %RET is dominated by sex (~75%, with males > females). On the other hand, MUT RET and MUT RBC variation is mainly attributable to inter‐animal variation (~72–91%), with much lower contributions from sex (~2–8%) and study number (~8–21%)

Figure 1 illustrates a set of historical negative control data (*n* = 39 male and female Crl:CD[SD] rats) derived from 13 separate studies conducted over 14 months. As described in the original report (Dertinger *et al*., 2019), these rats were exposed to one of several common vehicles and were 7 weeks old at time of blood collection. Restricted Maximum Likelihood (REML) analysis was conducted to evaluate the degree to which sex, study number, and inter‐animal differences contributed to the variation in %RET and MUT RET/RBC frequencies (Corbeil and Searle, 1976). As shown by Figure 1, variation in %RET is dominated by sex (~75%; males > females). On the other hand, MUT RET/RBC variation is mainly attributable to inter‐animal variation (~72–91%), with much lower contributions from sex (~2–8%) and study number (~8–21%).

## 3 EXPERIMENTAL DESIGN

### 3.1 Animal considerations

The *Pig‐a* assay has been performed most often with several commonly used laboratory strains of rat, including Sprague Dawley, Wistar Han, and Fischer 344 (Shemansky *et al*., 2019). Other rodent strains or species (including, e.g., transgenic mice) may be used provided they are responsive to known mutagenic agents, and historical negative control databases have been established as described above. Rats should be 4‐ to 10‐weeks old when dosing begins. Animals outside of this age range can be used, if appropriately justified. Animals are randomly assigned to negative control and test substance dose groups and should be uniquely identified after acclimatization to laboratory conditions for at least 3 days (or as prescribed by the applicable IACUC or their equivalent). Before randomized group assignment, it is recommended that an individual rat's body weight does not exceed ±20% of the group mean weight (sexes considered separately), and if a pre‐dosing assay is conducted, acceptable *Pig‐a* mutant cell frequencies may be used as a requisite for placement on study (discussed below).

The *Pig‐a* assay can be performed in either sex; the majority of published rodent *Pig‐a* studies, however, have utilized only males. In the case of single sex rat studies, at least six animals should be randomly assigned to each treatment group (Dertinger *et al*., 2011a; Gollapudi *et al*., 2015; OECD, 2020a). Whereas the goal is to have six analyzable rats per treatment group at the end of the study, if for unforeseen circumstances five animals remain in one or more treatment groups, the study is still considered valid (Gollapudi *et al*., 2015). Generally, at least four dose groups will be necessary: concurrent vehicle control, and low, mid and high dose groups. If data are not available to set appropriate dose levels, it is recommended first to perform a dose range finding study to select the maximum tolerated dose, maximum feasible dose, or determine the appropriateness of the regulatory limit dose (i.e., 1,000 mg/kg/day when dosing is conducted for ≥14 consecutive days). As with other in vivo genotoxicity studies, lower doses are generally separated by a factor of 2–3. If lower dose levels are necessary, for instance when the *Pig‐a* endpoint is being integrated into a 28‐day repeat‐dose toxicology study that is attempting to find a no observed adverse effect level or a benchmark dose, it will often be preferable to add additional dose group(s) as opposed to relying on very wide dose spacing.

If there are data indicating a difference in a test substance's toxicity, metabolism, or bioavailability between males and females, both sexes should be studied. Furthermore, it is also important to recognize that initiatives are underway to increase the number of preclinical and clinical studies that consider sex as a biological variable (NIH, 2015; Miller *et al*., 2017). It is therefore conceivable that over time more safety assessment studies will include both sexes. When both sexes are studied, equal numbers should be used in each treatment group. For studies that require different dose levels for males and females, the number of animal/sex/group will be similar to the single sex studies: that is, at least six males and six females per group, with a target of five per sex at the end of the study. For studies that treat animals of both sexes with the same dose levels, it is possible to reduce the number of animals per group. In these cases, it is useful to take advantage of factorial statistical designs which help maintain statistical power while limiting animal use. The requirement for proficiency demonstrations as described above, coupled with 3Rs principles and the desirability of integrating the *Pig‐a* assay within other toxicity tests, means that concurrent positive control animals are not ordinarily required. However, when laboratories are gaining experience with the *Pig‐a* assay, or for other reason(s) desire concurrent positive control rodents in their studies, it is not necessary to treat positive control animals using the same route of exposure, same vehicle, same treatment schedule, or on the same days that study animals are dosed. The latter design consideration takes advantage of the persistence of elevated MUT RET/RBC in circulation following exposure to mutagenic substances. For instance, one efficient and effective scenario is to expose positive control rats to ENU (e.g., 20 mg ENU/kg/day via oral gavage) on study Days 1, 2, and 3. Blood can then be collected from these animals much later, that is, at the same time blood samples from study animals that were treated over the course of several weeks are harvested (as described in more detail, below).

### 3.2 Treatment and blood sampling schedule

A 28‐day repeat‐dose protocol is preferred for conducting the *Pig‐a* assay for regulatory safety assessments. Of the dosing schedules evaluated to date, the 28‐day repeat‐dose schedule offers the most compelling evidence that a negative (non‐mutagenic) test result is reliable (OECD, 2020a; 2020b). Other repeat‐dose protocols may also be acceptable, if scientifically justified. There may also be instances when an acute dosing regimen is preferable, for instance when certain other genotoxicity endpoints are included in the experiment, and/or when there is a desire to maximize the cumulative test substance dose or plasma levels, *albeit* for a short time, as opposed to total exposure over a more extended period of time (Roberts *et al*., 2016). Whenever an acute treatment schedule is employed, it must be scientifically justified, and it is important to take the expression time of the MUT RET/RBC into account, which generally means delaying blood sample collection time(s) for two or more weeks.

Regardless of the dosing schedule, it will often be advantageous to perform *Pig‐a* analyses prior to the first administration of test substance (i.e., baseline samples taken within 1 week of dose initiation). As indicated above, this facilitates removal of rare “jackpot” animals from study that exhibit unusually high spontaneous MUT RET and/or MUT RBC frequencies. The utility of baseline analyses was foreseen by a renowned geneticist and mutagenesis expert who explained to one of the authors (SDD) when rodent blood‐based *Pig‐a* assays were beginning to be investigated: “You should be prepared to deal with outliers from the beginning for it is in the nature of spontaneous mutations to be clonal—non‐mutants are also clonal, but are invisible as clones” (Dr. John Heddle, Professor Emeritus, York University, July 4, 2008).

When animals are exposed to a test substance using the preferred treatment schedule, 28 consecutive days, at least one post‐exposure blood sample should be collected from each animal within day(s) of exposure cessation (e.g., Days 29–31; where “Day 1” is the day treatment begins). Data collected from testing diverse genotoxicants suggest that this is sufficient time for adequate manifestation of MUT RET/RBC responses and that the exact timing of sample collection within the suggested time window is not critical (OECD, 2020a; 2020b). This schedule has the advantage of facilitating integration of the *Pig‐a* assay within commonly utilized general toxicity and other genetic toxicology study designs. Accordingly, the test substance may be administered via one of the standard rodent routes: oral (gavage, diet, or drinking water), subcutaneous, inhalation, or intravenous; non‐standard routes can be used when scientifically justified. Note that an extra administration of test substance on Day 29, followed by sample collection several hours later, is permissible for accommodating tissue harvest when integrating the in vivo comet assay.

While additional time points are not required, there are certain opportunities to conduct *Pig‐a* analyses on blood samples collected at later time points. For instance, some toxicology experiments include “recovery” or “withdrawal” groups to evaluate whether toxic effects diminish, resolve, or increase upon discontinuation of dose. Blood samples from such animals are typically collected between 2 and 4 weeks after cessation of dosing and represent another opportunity to evaluate MUT RET/RBC (and provides additional time for manifestation of MUT RET/RBC induced by the doses administered later in the study).

## 4 BLOOD HARVEST, STORAGE, AND TRANSPORTATION

Applying animal welfare standards that minimize discomfort and stress, small volumes of peripheral blood can be obtained using a method that permits survival of the animal, such as bleeding from the tail vein, jugular vein, or other appropriate blood vessel. Alternately, immediately after animals are sacrificed, blood can be collected via cardiac puncture or sampling from a large blood vessel (abdominal aorta or vena cava). As flow cytometric analysis requires single cell suspensions, care must be taken to avoid blood coagulation. This is normally accomplished using an anticoagulant, such as heparin and/or EDTA. It is good practice to collect at least two‐times more blood than is necessary for the *Pig‐a* assay. This represents a back‐up that can be useful for myriad reasons that include a labeling/technical issue that compromises the mutant analysis, shipment failures (delayed/lost/damaged during transit), or when a determination has been made that more cells need to be analyzed.

In the presence of anticoagulant, blood samples can be stored for up to 5 days before they are processed for flow cytometric analysis as long as they are maintained cold, but not frozen (e.g., in a 4°C refrigerator; Gollapudi *et al*., 2015). Furthermore, anticoagulant‐treated blood samples can be shipped to an analytical facility provided they are maintained cold throughout transportation and any subsequent storage, and as long as they are further processed/analyzed within 5 days of collection (Gollapudi *et al*., 2015).

Procedures also have been described for freezing and later thawing blood samples for subsequent processing and flow cytometric analysis of *Pig‐a* MUT RET/RBC frequencies (Avlasevich *et al*., 2019). These procedures can be useful for delaying analysis for reasons that include instrument failure, deferring the decision to acquire *Pig‐a* data, and storing blood from mutagen‐treated animals for use as analytical positive control samples. Furthermore, frozen blood samples can be transported from an in‐life site to an analytical site provided they are maintained frozen throughout transportation (e.g., on dry ice) (Avlasevich *et al*., 2019). Whatever freezing and thawing method is employed, it is important to demonstrate minimal lysis of RBCs, and that the freezing and thawing process, and length of storage, have minimal impact on MUT RET/RBC and RET frequencies.

## 5 SYSTEMIC EXPOSURE

A negative in vivo *Pig‐a* test result will carry no weight unless evidence is provided that the bone marrow was exposed to the test substance. With toxic compounds, evidence of bone marrow exposure can be demonstrated by significant changes to the percentage of reticulocytes in peripheral blood circulation. This will usually be seen as reduction in %RET when blood is collected within hours to day(s) of treatment cessation. However, when blood is collected several days or more after discontinuing treatment, it can manifest as elevated %RET frequencies due to stress erythropoiesis. (Note that Nicolette *et al*., 2018 demonstrated that regenerative erythropoietic response does not increase the frequency of MUT RET/RBC in rats.) Test substance‐induced hemolysis is another situation that can manifest as elevated frequencies of reticulocytes and represents evidence of systemic exposure (Kenyon *et al*., 2015). In the absence of toxicity to the erythropoietic system, other evidence can be provided by concomitantly measuring plasma or blood levels of the test substance and/or its metabolites, since bone marrow is extremely well‐perfused (Marenzana and Arnett, 2013; EFSA, 2017; Grüneboom *et al*., 2019). ADME (absorption, distribution, metabolism, and excretion) data, obtained in an independent *(*i.e., a separate) study using the same dosing route and same species, may also be used to demonstrate bone marrow exposure. Another way to ensure systemic exposure for chemicals with in vitro genotoxic activity and low likelihood of reaching the bone marrow due to chemical reactivity is to administer the test substance intravenously. This was recently done to support a negative *Pig‐a* finding in an aryl boronic acid study (Masuda‐Herrera *et al*., 2019).

## 6 DATA ACQUISITION

### 6.1 Flow cytometry

Flow cytometric analysis is the analytical method of choice for determining circulating RET and MUT RET/RBC frequencies. The preferred antibodies used to prepare erythrocytes for flow cytometric analysis are anti‐CD59 for rats and anti‐CD24 for mice (Gollapudi *et al*., 2015; OECD, 2020a). Other GPI‐anchored proteins exist on the surface of wild‐type RBCs (e.g., CD55), and antibodies against such surface markers can be used if sufficiently validated. Additionally, it is possible to use combinations of antibodies to distinguish wild‐type from mutant phenotype cells (e.g., anti‐CD59 and anti‐CD55). However, a single antibody against the highly expressed CD59 and CD24 surface markers are sufficient for assaying *Pig‐a* mutant frequencies in rats and mice, respectively.

When a positive control group is not included in a study, a “mutant mimic” or comparable sample should be used to demonstrate the light scatter and fluorescence characteristics of wild‐type versus MUT RET/RBC. Mutant mimics can be created by processing extra blood from a vehicle control animal and omitting the fluorescent GPI‐anchored antibody(s) from the labeling protocol (Phonethepswath *et al*., 2010; Raschke *et al*., 2016). Since mutant mimics are valuable for guiding instrumentation settings and software/data analysis parameters, they should be generated for every study, and used each day blood samples are analyzed.

An alternative to mutant mimics is to use blood samples previously collected from mutagen‐dosed (positive control) animals. Such samples can be stored frozen (as described above) and used to identify the light scatter and fluorescence characteristics of wild‐type versus MUT RET/RBC. In these cases, it is usually sufficient to include 1–3 such blood samples each day of analysis. When these samples are being used in place of mutant mimics, the positive control blood sample(s) should demonstrate levels of MUT RET/RBC that are elevated sufficiently to establish the fluorescence characteristics of mutant phenotype cells. For this purpose, it is ideal for the mutant frequency in these two cell populations be at least 100 mutant cells per million erythrocytes.

### 6.2 Number of cells evaluated

According to industry best practices, as well as the IWGT *Pig‐a* expert report and *Pig‐a* Detailed Review Paper, the minimum number of RBC and RET that should be evaluated for the *Pig‐a* mutant phenotype per animal and per time point is 1 × 10^6^ (Gollapudi *et al*., 2015; OECD, 2020a). This was the minimum number of cells analyzed for each of the chemicals included in the retrospective validation report (OECD, 2020a; 2020b). Therefore, analyzing 1 × 10^6^ RET and RBC for *Pig‐a* mutation has been shown to be effective at detecting mutagenic test substances.

While 1 × 10^6^ cells analyzed per animal and per time point has been a widely cited minimum, it is important to keep analyses that return zero (0 × 10^−6^) mutant cell frequency readings to an occasional, rather than common, occurrence. Proficient laboratories have shown that for commonly used rodent models, mean baseline MUT RET/RBC frequencies are on the order of 1–3 × 10^−6^. Given this information, it should not be surprising that it may be necessary to evaluate more than 1 × 10^6^ cells in order to avoid a high prevalence of zero MUT RET/RBC frequency readings. This decision about number of cells evaluated per animal per time point is ideally made as the laboratory develops their historical negative control database. This represents the best time to set the number of cells evaluated in a data‐driven manner and is greatly preferred to relying on the cited minimum value of 1 × 10^6^ cells and having to defend study results that exhibit a high prevalence of zero readings.

Finally, given the rarity of RET in peripheral blood circulation, it is not practical to evaluate ≥1 × 10^6^ RET directly from blood samples. In order to overcome this problem, immunomagnetic separation procedures prior to flow cytometric analysis were developed to increase the number of RET (and in some cases the number of RBC) interrogated for MUT RET/RBC measurements (Kimoto *et al*., 2011a; Dertinger *et al*., 2011b; Chickura *et al.,* 2021). These immunomagnetic separation techniques, or a validated alternative, are a practical solution for evaluating adequate numbers of cells as described above.

## 7 STATISTICAL CONSIDERATIONS

Statistical analysis of biological data can be an area of contention. There is no single correct method of conducting a statistical analysis, and statisticians can differ in their preferred methodology. Some of these differences are fundamental and deeply philosophical such as between Bayesians and Frequentists. There is considerable concern by a large proportion of statisticians at the continuing use of *p*‐values and statistical significance in the interpretation of results. Increasingly, there is a preference for estimates of the size of effects with confidence intervals to be evaluated in preference to *p*‐values. (Bayesians have a different viewpoint on this as well). Linked to this is the greater emphasis on a modeling approach to data analysis brought about in part by the developments in statistical theory and the availability of much greater computing power. This can create a clash between the expectation for modern methods to be used against the use of methods that are based upon approaches which were developed in the pre‐personal computer area and based upon algorithms and methods which were, in effect, short‐cuts or work arounds to the analysis. It also can complicate the task of those seeking a simple “yes/no” result from an experiment.

A practical approach is to suggest a particular set of statistical analyses as an example of the sort of analyses that can be carried out. It should be made clear that this is not a prescribed method and may not be suitable for all sets of data. It would be quite acceptable for someone to use an alternative method, especially if our suggested method is not considered suitable. However, they must be prepared to justify their approach.

### 7.1 Data analyses

One set of statistical tests are pairwise comparisons of MUT RET/RBC and RET frequencies in the concurrent vehicle control group with those measured in the test substance exposed groups. Parametric analyses such as ANOVA with post hoc multiple comparison tests are commonly used, but other methodologies are equally acceptable. Generally, these types of parametric tests should be performed only when assumptions such as normality of the distribution within, and homogeneity of variance among groups are confirmed (e.g., using tests such as, Levene's and/or Brown‐Forsynthe tests). If heteroscedasticity is identified, an appropriate data transformation such as a logarithmic (log_10_) can be used. Note that if there are animals with 0 (zero) mutant frequency values, a small constant offset value such as 0.1 should be added to *every* animal's mutant cell frequency before transformation because log_10_ of zero is “not defined” and will prevent calculations. If the transformation does not restore homoscedasticity, non‐parametric pairwise comparison methods may be considered, for example the Kruskal‐Wallis test and post hoc Dunn's test. These methods can be extended to other experimental designs, such as the factorial design, where both treatment and sex are factors in the analysis.

A related statistical test described in current OECD in vivo genotoxicity guidelines is a trend test to identify a dose–response relationship. Care is needed in interpreting the results of some trend tests, for instance a simple linear trend test, because they may fail to detect a trend when, for instance, the dose–response is non‐monotonic. Trend tests capable of detecting non‐monotonicity such as the downturn protection test proposed by Bretz and Hothorn (2003), may be useful in such cases.

The third analysis considers whether the mean MUT RET and/or MUT RBC frequency of any test substance treatment group exceeds the upper bounds of the historical negative control data distribution. As discussed previously, there are several valid approaches for characterizing the distribution of historical negative control data, including prediction intervals, tolerance intervals, and control limits. In the field of Quality Control, control limits are defined as lines plotted on a control chart 3 *SD*s above and below the mean. Each laboratory must define an appropriate limit based on their data. In some instances, it may be appropriate to use a different interval, for example the 95% reference interval (mean ±1.96 *SD*s). Also note that comparing group means to an upper bound limit value derived from an appropriate historical negative control distribution is not the only comparison that can be made. For instance, it could also be useful to consider the relationship of individual animal's mutant cell frequencies to the historical negative control upper bound limit value when a single rodent is so highly elevated that it is responsible for the elevated group mean.

### 7.2 Interpretation of results

**FIGURE 2:**
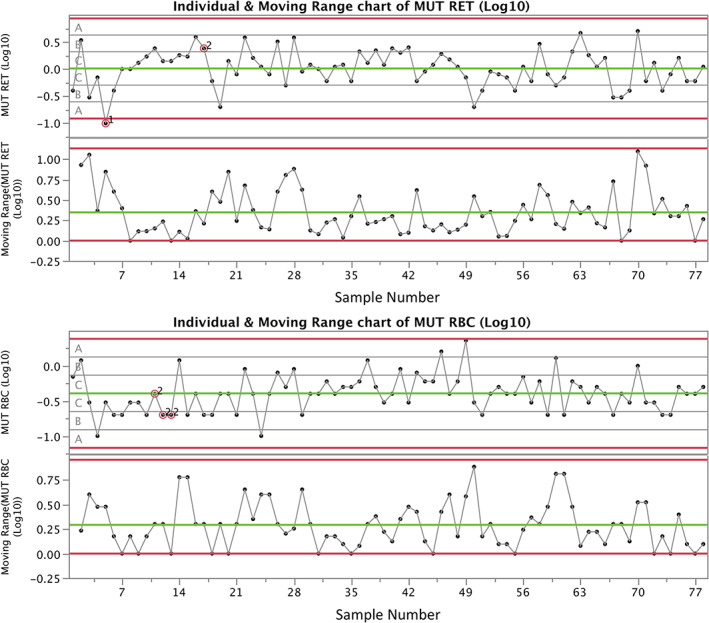
Mutant reticulocyte (MUT RET) and mutant erythrocyte (MUT RBC) frequencies are graphed for the same vehicle‐exposed male and female rats portrayed in Figure 1 (i.e., 78 individuals). In this case, the results are plotted on control charts according to the order that analyses occurred (13 studies over 14 months). Zones A, B, and C signify values that are within 3, 2, and 1 *SD* from the mean, respectively. Nelson rules violations (numbered 1–8) are superimposed on data points when alerts are triggered. In the current example, “1” indicates a value is greater than 3 *SD*s from the mean, while “2” signifies that nine or more points in a row are on the same side of the mean. Overall, the low number of violations gives one confidence that the mutant cell scoring process is “under control.” While these charts were produced using the JMP software (v12.0.1), other packages such as Minitab can produce similar charts, as can packages in the R statistical programming language

When assessing *Pig‐a* results, the study must first be deemed valid. This includes, in part, mean concurrent vehicle control treatment group MUT RET/RBC frequencies that are below the upper limit of the negative historical control distribution and are technically uncompromised. This also includes some demonstration that the historical negative control database is of sufficient quality to provide a reasonable assessment of those responses that exceed its distribution bounds. One recommended method for assessing the quality of the historical control database is the use of control charts in conjunction with Nelson rules (Nelson, 1984; see Figure 2). Other factors such as the number of animals evaluated per group, instances of zeros in the dataset, and sample quality should be consistent with the guidance given above.

When evaluating whether the test substance induced increases in MUT RET/RBC frequencies, the analytical approaches described above are regarded as key tools. Positive test substances will result in the aforementioned three elements (significant pairwise comparison, significant trend increase, test substance response greater than the historical negative control distribution) aligning with each other. Negative test substances will produce data that are not consistent with any of the three elements used for consideration. Scientific judgment will be essential in those cases where they are not all in agreement. This paradigm is reinforced by an expert OECD genotoxicity working group that concluded “…data should be interpreted based both on statistics and biological relevance” (OECD, 2016).

In certain instances, even after applying expert judgment, it will not be possible to classify a response as positive or negative. In these cases the response is equivocal and further testing may be required to resolve the mutagenicity of the test substance. This is obviously not as straight‐forward as conducting statistical tests and referring to an immutable rubric to make final judgments, but it is considered the best scientific approach according to the aforementioned expert working group (OECD, 2016).

## 8 CLOSING THOUGHTS

The *Pig‐a* assay represents an efficient means for studying the potential of chemicals to induce mutation in vivo in hematopoietic cells. The need for systemic availability of the test substance, coupled with knowledge about the kinetics by which MUT RET and MUT RBC appear in peripheral blood circulation, are the main determinants for good experimental design and interpretation. The other critical factor is analytical proficiency, which when demonstrated as described, will generate a useful negative historical control distribution that is invaluable for assessing assay acceptability and assay responses. We hope the recommendations provided herein will be helpful to the genetic toxicology safety assessment community.

## DISCLOSURE OF INTERESTS

JB, DR, LFS Jr, and LR are employed at contract research organizations that offer rodent blood‐based *Pig‐a* assay testing services. SD is employed by Litron Laboratories; Litron holds patents for enumerating *Pig‐a* mutant phenotype erythrocytes and sells kits and offers blood sample analysis services based on these methods. The views expressed herein are the authors', and do not necessarily reflect the views or official positions or policies of the NIEHS, the U.S. Food and Drug Administration, or any of the other institutions and businesses associated with the authors.

## AUTHOR CONTRIBUTIONS

Stephen D. Dertinger, Javed A. Bhalli, and Daniel J. Roberts conceived and outlined the article, and wrote the first draft. All co‐authors contributed to the extensive revisions and edits that followed.
